# Modified Gray-Level Coding Method for Absolute Phase Retrieval

**DOI:** 10.3390/s17102383

**Published:** 2017-10-19

**Authors:** Xiangcheng Chen, Shunping Chen, Jie Luo, Mengchao Ma, Yuwei Wang, Yajun Wang, Lei Chen

**Affiliations:** 1 School of Automation, Wuhan University of Technology, Wuhan 430070, China; chenxgcg@ustc.edu (X.C.); luo_jie@whut.edu.cn (J.L.); 2 Department of Precision Machinery and Precision Instrumentation, University of Science and Technology of China, Hefei 230026, China; shun3568@mail.ustc.edu.cn; 3 Department of Instrument Science and Opto-Electronics Engineering, Hefei University of Technology, Hefei 230088, China; mmchao@hfut.edu.cn; 4 State Key Laboratory of Information Engineering in Surveying, Mapping, and Remote Sensing, Wuhan University, Wuhan 430079, China; 5 School of Mechanical and Electrical Engineering, Wuhan University of Technology, Wuhan 430070, China; chenlei811001@163.com

**Keywords:** fringe projection, phase retrieval, phase shift, gray-level coding

## Abstract

Fringe projection systems have been widely applied in three-dimensional (3D) shape measurements. One of the important issues is how to retrieve the absolute phase. This paper presents a modified gray-level coding method for absolute phase retrieval. Specifically, two groups of fringe patterns are projected onto the measured objects, including three phase-shift patterns for the wrapped phase, and three *n*-ary gray-level (*n*GL) patterns for the fringe order. Compared with the binary gray-level (*b*GL) method which just uses two intensity values, the *n*GL method can generate many more unique codewords with multiple intensity values. With assistance from the average intensity and modulation of phase-shift patterns, the intensities of *n*GL patterns are normalized to deal with ambient light and surface contrast. To reduce the codeword detection errors caused by camera/projector defocus, *n*GL patterns are designed as *n*-ary gray-code (*n*GC) patterns to ensure that at most, one code changes at each point. Experiments verify the robustness and effectiveness of the proposed method to measure isolated objects with complex surfaces.

## 1. Introduction

Optical three dimensional (3D) sensing systems are becoming increasingly used in many fields such as medical sciences, industrial inspection and virtual reality. Among various technologies, digital fringe projection (DFP) has been a major research subject in terms of speed, accuracy, resolution, and ease of use [1,2,3,4,5]. In a typical DFP system, some pre-designed patterns are projected onto the measured objects by a projector from one viewpoint, and modulated by the objects’ profiles. Meanwhile, their corresponding deformed images are captured by a camera from another viewpoint. Then the phase modulation can be calculated through suitable digital fringe analysis. Finally, the DFP system should be calibrated to recover the relationship between the phase modulation and the real 3D world coordinates [6,7,8]. Therefore, the phase extraction accuracy will directly affect the 3D shape measurement result [9].

Common projected patterns include sinusoidal [10], binary [11], triangular [12], trapezoidal [13], etc. Instead of gray images, color images containing red, green and blue channels may be employed for fast measurement, but this method is sensitive to system noise, nonlinearity and color coupling [14,15,16,17]. The binary, triangular or trapezoidal patterns become sinusoidal in shape when they are blurred to a certain level [18]. Therefore, it would seem reasonable to adopt sinusoidal patterns directly. The methods used to demodulate 3D information from fringe patterns are referred to as digital fringe analysis. Fourier transform [19], windowed Fourier transform [20], wavelet transform [21] and phase-shift [22] are common digital fringe analysis algorithms. Among them, phase-shift methods have been extensively studied due to their pixel-by-pixel measurement [23]. However, the wrapped phase calculated directly from the arctangent function has 2π phase jumps which should be unwrapped to obtain the absolute phase. Ideally, those 2π phase jumps can be easily removed with reference to the neighboring pixels. However, the neighboring pixels are often invalid because of local shadows and isolated objects that result in some challenges for phase unwrapping [24].

The existing phase unwrapping algorithms can be divided into two principal categories: spatial algorithms and temporal algorithms [25]. Spatial algorithms have been investigated with a variety of different considerations [26,27,28]. Spatial algorithms tend to fail when dealing with discontinuous or isolated objects therefore, researchers have focused on the more efficient temporal algorithms which can avoid the error transferring between pixels [29]. Up until now, several temporal algorithms have been developed to address this problem, such as two-wavelength [30], multiple-wavelength [31], gray-code [32], and phase-coding [33]. Among them, the gray-code method is a simple, commonly used *b*GL method, in which a unique codeword is assigned to each fringe period. As only two intensity values are employed, thus *m* patterns can generate 2*^m^* codewords. A large number of patterns slows down the measurement speed, and makes this method unsuitable for high-speed measurements. The *n*GL method which encodes *n* > 2 intensity values effectively reduces the number of coded patterns, thus *m* patterns can generate *n^m^* codewords. For example, if *n* = 4, and *m* = 3, the *b*GL method can generate 2^3^ = 8 codewords, in contrast the *n*GL method can generate 4^3^ = 64 codewords which is many more than the former method. Instead of directly using the gray-level patterns for the shape measurement [5], the *n*GL patterns are only used for fringe order calculations in this paper. Meanwhile, gray-level methods [34,35,36,37] employ an additional pattern for uniform illumination, then intensity ratios which encode the spatial locations are calculated. In contrast, the *n*GL method does not need uniform illumination for background calculation.

However, ambient light and surface contrast make codeword detection difficult in the *n*GL method. To overcome this problem, the average intensity and modulation of phase-shift fringes are employed to eliminate these influences. In addition, the codes change at the same place in *n*GL patterns, which creates a tendency for codeword detection errors at the code boundaries, especially for blurred patterns [38]. Caspi [39] used a generalized Gray code method [40] for range imaging with color structured light, which has the same advantage as the binary Gray code method [32]. That is, the effect of a detection error due to a transition is limited to an error between the two adjacent light planes. Horn [37] and Porras-Aguilar [36] proposed the use of projection patterns where two neighboring gray-levels differ only by one grey-level to avoid the detection error at code-boundaries, which is known as the Hamming distance of 1 gray-level. Furthermore, Porras-Aguilar [34] demonstrated an optimal coding scheme avoiding the influence of defocus errors using space-filling curves, so that the codewords can have one or two gray-level changes. In this paper, the coded patterns were only used for identifying the fringe orders. A *n*-ary gray-code method, similar to Caspi [39], was used to reduce the codeword detection errors at the code boundaries, whereby at most, one code changes at each point for different coded patterns. Using the proposed method, the codeword can be detected more accurately, and the phase retrieval performance can be improved.

The remainder of the paper is organized as follows: Section 2 presents the principles of the proposed method in detail; Section 3 and Section 4 demonstrate our method through simulation and experiment, respectively, and finally, Section 5 summarizes this research.

## 2. Materials and Methods

### 2.1. Three-Step Phase-Shift Method

Phase-shift methods have been widely used in optical metrology because of their speed and accuracy [41]. Among various phase-shift methods, the three-step method requires the least number of patterns for phase recovery. Three sinusoidal phase-shift fringe patterns with equal phase shifts captured by camera can be mathematically described as:

(1) $$I_{1}{}^{c}(x,y)=A^{c}(x,y)+B^{c}(x,y)\cos\left[\phi (x,y)-2\pi /3\right]$$

(2) $$I_{2}{}^{c}(x,y)=A^{c}(x,y)+B^{c}(x,y)\cos\left[\phi (x,y)\right]$$

(3) $$I_{3}{}^{c}(x,y)=A^{c}(x,y)+B^{c}(x,y)\cos\left[\phi (x,y)+2\pi /3\right]$$

where $A^{c}(x,y)$ denotes the average intensity, B(x,y)c denotes the intensity modulation, and ϕ(x,y) denotes the modulating phase to be solved. Combining the above equations, the three variables can be calculated as [42,43,44]:

(4) $$A^{c}(x,y)=(I_{1}{}^{c}+I_{2}{}^{c}+I_{3}{}^{c})/3$$

(5) $$B^{c}(x,y)=\sqrt{{\left(I_{1}{}^{c}-I_{3}{}^{c}\right)}^{2}/3+{\left(2I_{2}{}^{c}-I_{1}{}^{c}-I_{3}{}^{c}\right)}^{2}/9}$$

(6) $$\phi (x,y)=\arctan\left(\sqrt{3}\frac{I_{1}{}^{c}-I_{3}{}^{c}}{2I_{2}{}^{c}-I_{1}{}^{c}-I_{3}{}^{c}}\right)$$

Generally, background and shadow regions can be removed with the assistance of the modulation map and a segmentation threshold. Due to the arctangent operation, the solved phase map will be limited in range of [−π, π] with 2π discontinuities. Thus, phase unwrapping should be carried out to remove these discontinuities. The key to the phase unwrapping is to determine the fringe orders. If the fringe orders k(x,y) are determined, the wrapped phase can be unwrapped as:

(7) Φ(x,y)=ϕ(x,y)+k(x,y)×2π

where Φ(x,y) denotes the unwrapped phase or absolute phase.

### 2.2. Intensity Normalization for Coded Patterns

In this paper, we employ extra coded patterns to calculate the fringe orders. Three coded patterns used to encode the codewords can be mathematically described as:

(8) $$J_{1}{}^{c}(x,y)=A^{c}(x,y)+B^{c}(x,y)\times \alpha_{1}\left(x,y\right)$$

(9) $$J_{2}{}^{c}(x,y)=A^{c}(x,y)+B^{c}(x,y)\times \alpha_{2}\left(x,y\right)$$

(10) $$J_{3}{}^{c}(x,y)=A^{c}(x,y)+B^{c}(x,y)\times \alpha_{3}\left(x,y\right)$$

Similarly, $A^{c}(x,y)$ denotes the average intensity, $B^{c}(x,y)$ denotes the intensity modulation, $\alpha_{1}\left(x,y\right)$ , $\alpha_{2}\left(x,y\right)$ and $\alpha_{3}\left(x,y\right)$ denote the coded coefficients ranging from −1 to 1. Note that, $A^{c}(x,y)$ and $B^{c}(x,y)$ are assigned the same values as that of phase-shift patterns, thus they can be computed with Equations (4) and (5). The following equations used to normalize the coded patterns can be described as:

(11) $$\alpha_{1}=\left(J_{1}{}^{c}-A^{c}\right)/B^{c}$$

(12) $$\alpha_{2}=\left(J_{2}{}^{c}-A^{c}\right)/B^{c}$$

(13) $$\alpha_{3}=\left(J_{3}{}^{c}-A^{c}\right)/B^{c}$$

Through the above equations which take the average intensity and modulation into consideration, the influences of ambient light and surface contrast can be eliminated, and the codes can be exactly identified.

### 2.3. The n-Ary Gray-Code Method

**Figure 1:**
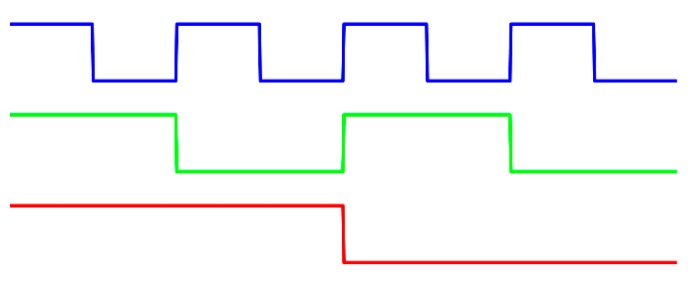
The binary gray-level (*b*GL) method.

Among the various temporal phase unwrapping algorithms, the *b*GL method may be the simplest way to resolve phase ambiguity [45]. In this method, codewords are encoded within binary patterns used to mark the fringe orders of the phase-shift patterns. Figure 1 shows three-frame binary patterns as an example. There are two intensity values: the black stripes are assigned to the logical value 0, while the white stripes are assigned to the logical value 1. In general, *m* patterns can generate 2*^m^* codewords, and each of them contains *m* bits. The pattern images can be sequentially captured by the camera. Then, the codewords of each pixel can be determined through suitable threshold algorithms. This method proves to be reliable and less sensitive to the surface contrast, since only binary values are used in all pixels. However, a larger number of patterns need to be projected to achieve high spatial resolution, which means that image acquisition takes too long and the measured objects have to remain static. Thus, this method is not suitable for real-time measurement [46].

**Figure 2:**
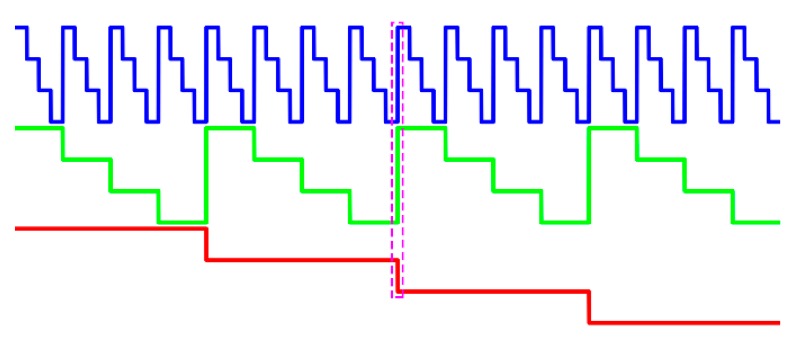
The *n*-ary gray-level (*n*GL) method.

To reduce the number of projected patterns, the *n*GL coding method is developed where more than two intensity values are encoded. Differing from the *b*GL method that only uses intensity values 0 and 255, the *n*GL method uses *n* > 2 intensity values from 0 to 255. Figure 2 shows the *n*GL method with *n* = 4 intensity levels. However, the images of *n*GL patterns become blurred due to the camera/projector defocus, which makes the codeword determination at code boundaries difficult. This problem will be worse if the codes change at the same place in different coded patterns.

**Figure 3:**
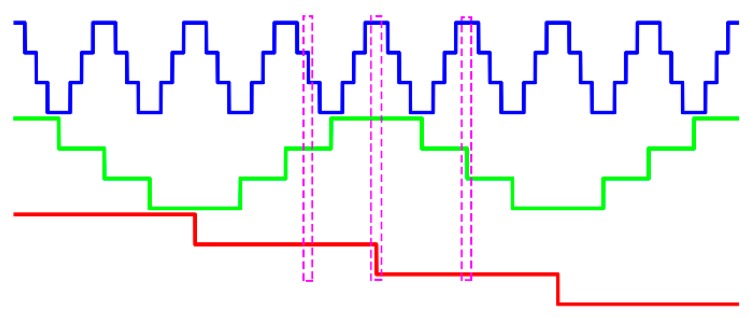
The *n*-ary gray-code (*n*GC) method used in this paper.

To tackle this problem, the *n*GC method is used to improve the conventional *n*GL method, as illustrated in Figure 3. Clearly, at most, one code changes at each pixel of all the *n*GC patterns. Moreover, the codewords do not appear more than once. The total number of codewords remains the same, yet the codeword detection errors occurring at the code boundaries could be reduced and the phase unwrapping can be improved.

**Figure 4:**
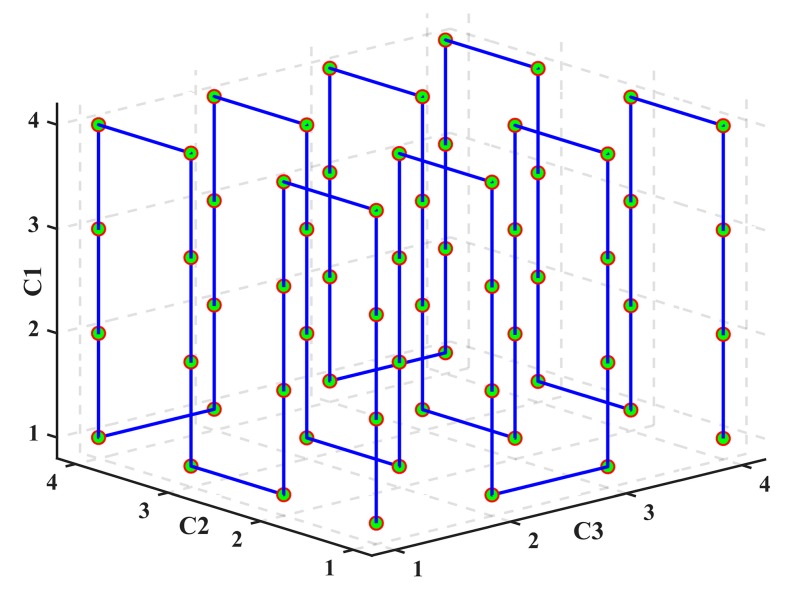
Code-space diagram.

In this paper, we use three *n*GC patterns with *n* = 4 intensity levels as an example. With these patterns, a total of 4^3^ = 64 codewords can be encoded, as illustrated in Table 1. Also, the codewords are drawn in code-space, as shown in Figure 4.

### 2.4. The Framework of the Proposed Method

The following steps describe the framework for absolute phase retrieval using the *n*GC method.

**Step 1: Design codewords.** Let *C*_1_, *C*_2_ and *C*_3_ be the code sequences for the three coded patterns. All designed 3-bit codewords are given in Table 1.
**Step 2: Calculate the code coefficients.** The code coefficients range from −1 to 1, while codewords range from 1 to 4. The following mathematical equations describe the mapping relationship from the codewords to the code coefficients.
(14) $$\alpha_{1}=C_{1}/2-5/4$$
(15) $$\alpha_{2}=C_{2}/2-5/4$$
(16) $$\alpha_{3}=C_{3}/2-5/4$$
**Step 3: Encode codewords into patterns.** With the three code sequences, the three coded patterns used to carry them can be mathematically described as:
(17) $$J_{1}{}^{p}(x,y)=A^{p}(x,y)+B^{p}(x,y)\times \alpha_{1}\left(k\right)$$
(18) $$J_{2}{}^{p}(x,y)=A^{p}(x,y)+B^{p}(x,y)\times \alpha_{2}\left(k\right)$$
(19) $$J_{3}{}^{p}(x,y)=A^{p}(x,y)+B^{p}(x,y)\times \alpha_{3}\left(k\right)$$
  where $A^{p}(x,y)$ and $B^{p}(x,y)$ are constants, k=⌈x/P⌉ denotes the fringe order; *P* denotes the number of pixels per fringe period.
**Step 4: Wrapped phase calculation.** Once the deformed phase-shift patterns are captured by the camera, $A^{c}(x,y)$ and $B^{c}(x,y)$ can be calculated on the basis of Equations (4) and (5), and the wrapped phase ϕ(x,y) can be calculated on the basis of Equation (6).
**Step 5: Intensity Normalization.** With the captured coded patterns, and $A^{c}(x,y)$ and $B^{c}(x,y)$ calculated in the previous step, $a_{1}(x,y)$ , $a_{2}(x,y)$ and $a_{3}(x,y)$ can be calculated on the basis of Equations (11)–(13).
**Step 6: Calculate codewords.** Then, *C*_1_, *C*_2_ and *C*_3_ can be obtained as:
  *C*_1_ = *Round*[2*a*_1_ + 5/2] (20)
  *C*_2_ = *Round*[2*a*_2_ + 5/2] (21)
  *C*_3_ = *Round*[2*a*_3_ + 5/2] (22)
  Here, *Round*(*x*) denotes the closest integer of input *x*.
**Step 7: Determine fringe order.** Looking at *C*_1_, *C*_2_ and *C*_3_ in Table 1, their order can be regarded as the fringe order. Then, we can convert the wrapped phase ϕ(x,y) to the absolute phase Φ(x,y) according to the Equation (7).

## 3. Simulations

**Figure 5:**
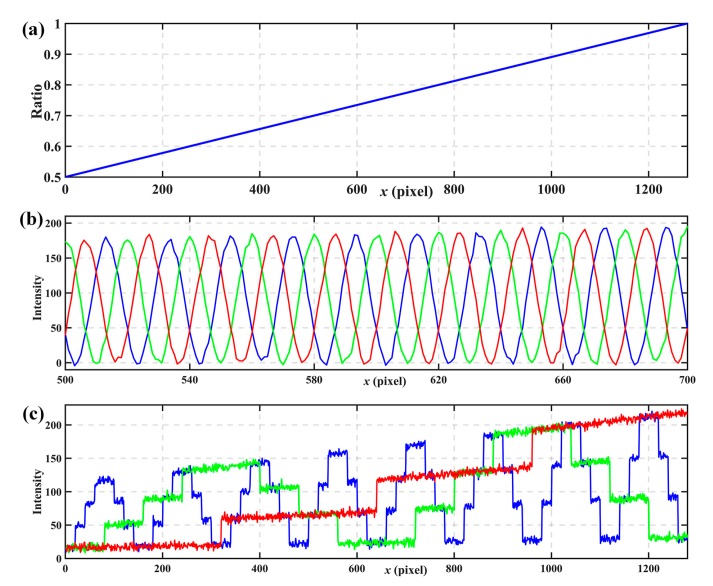
Simulated patterns. (**a**) modulation function, (**b**) modulated phase-shift patterns with noises, (**c**) modulated coded patterns with noises.

In order to explore the feasibility of the proposed method, some simulations have been done. Figure 5 shows the simulated phase-shift patterns and coded patterns. In those simulations, three *n*GC patterns are used to generate 64 codewords. The phase-shift patterns have a fringe period of *P* = 20 pixels. A modulation function is used to simulate variation of the background, as shown in Figure 5a, and the modulation function changes from 0.5 to 1.0. The function modulates both phase-shift fringe patterns and coded fringe patterns, and Gaussian noises are also added to the patterns. Figure 5b,c show the phase-shift fringe patterns and coded fringe patterns after modulating background intensity and adding Gaussian noises (10 dB). Figure 5c shows that it is difficult to differentiate the different codes from intensities because the background intensities change too much.

**Figure 6:**
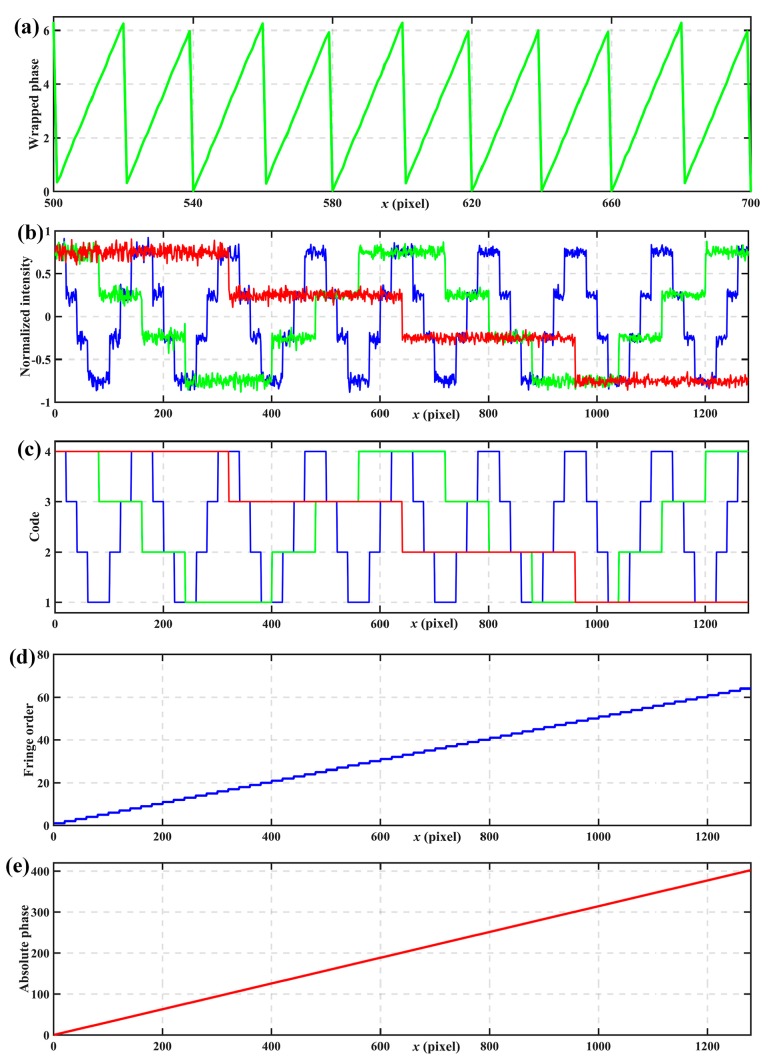
Simulated results. (**a**) wrapped phases, (**b**) coded patterns with intensity normalization, (**c**) codewords calculated from normalization coded patterns, (**d**) fringe orders, (**e**) the calculated absolute phase.

Figure 6 shows simulated results. Figure 6a shows the wrapped phases calculated on the basic of Equation (6). Figure 6b shows the coded fringe patterns with intensity normalization, where three thresholds, −0.5, 0, and 0.5 can divide the intensity into 4 levels unambiguously. Figure 6c shows the codes calculated from the normalization coded fringe patterns according to Equations (20)–(22). Figure 6d shows the fringe orders determined from Table 1. Figure 6e shows the calculated absolute phase according to Equation (7) after obtaining the wrapped phase and fringe orders.

## 4. Experiments

**Figure 7:**
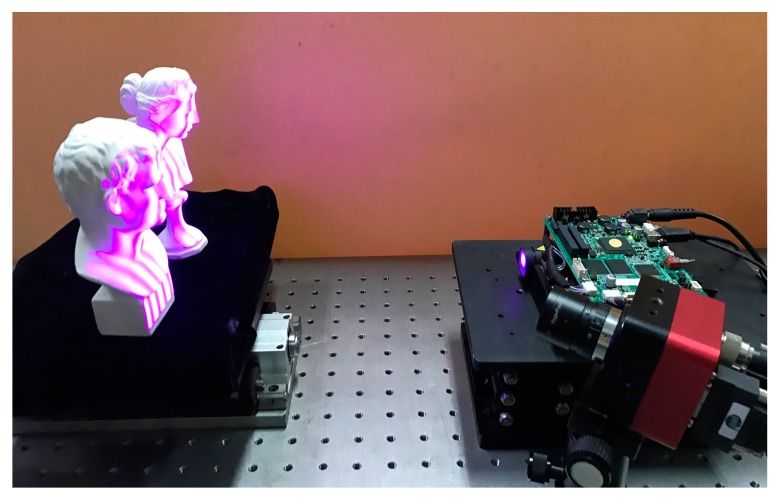
Setup used in the experiments.

To verify the performance of the proposed method, we developed a common fringe projection system including a COMS camera (IOI Flare 2M360-CL), a digital light processing projector (LightCrafter 4500) and a computer. Figure 7 shows the setup used in the experiments. The camera has a resolution of 1280 × 1024 pixels, and the images are delivered to the computer via high-speed Camera Link. The resolution of the projector is 912 × 1140 pixels. The measured objects are two separated plaster sculptures placed before the fringe projection measurement system. The sinusoidal phase-shift fringe patterns and *n*GC coded patterns were projected onto the measured objects by the projector sequentially, and then deformed fringe patterns were captured by the camera at the same time. For comparison, the *n*GL patterns were also projected and captured like the *n*GC coded patterns.

**Figure 8:**
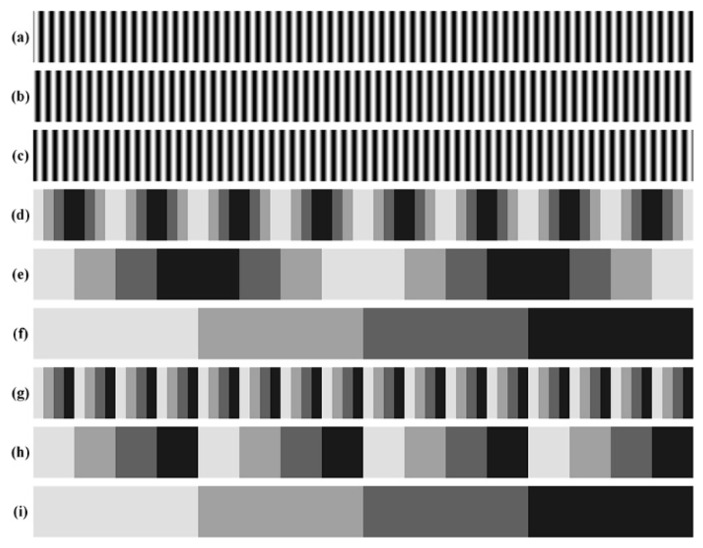
Projected patterns. (**a**–**c**) phase-shift patterns, (**d**–**f**) *n*GC patterns, (**g**–**i**) *n*GL patterns.

Figure 8 shows the projected patterns including three phase-shift patterns, three *n*GC patterns and three *n*GL patterns. Figure 8 shows the deformed images of these patterns, respectively. Figure 8a–c shows the images of phase-shift patterns, from which the wrapped phase can be calculated. Figure 8d–f shows the images of nGC patterns, from which the codewords representing the fringe order can be extracted. Figure 8g–i shows the *n*GL patterns for comparison.

**Figure 9:**
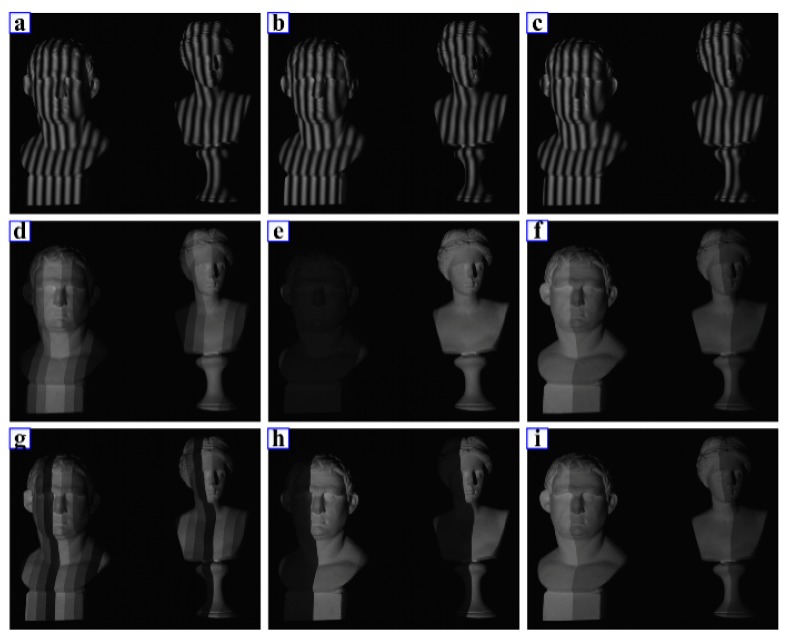
Captured images. (**a**–**c**) phase-shift patterns, (**d**–**f**) *n*GC patterns, (**g**–**i**) *n*GL patterns.

**Figure 10:**
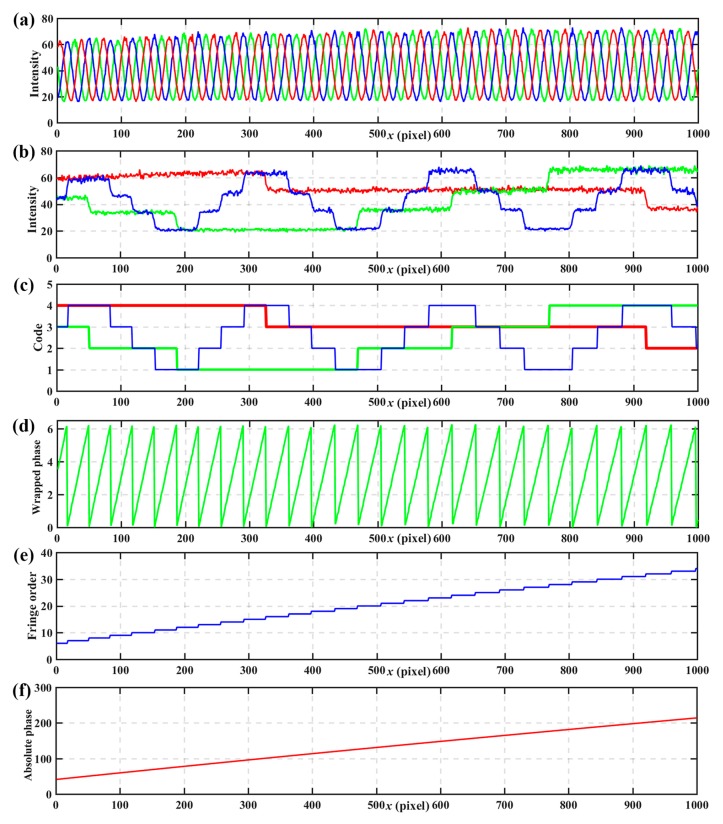
One cross-section of the reference plane. (**a**) phase-shift patterns, (**b**) *n*GC patterns, (**c**) calculated codewords, (**d**) wrapped phase, (**e**) determined fringe order, and (**f**) absolute phase.

Figure 9 shows the captured images of these projected patterns, including phase-shift patterns, *n*GC patterns and *n*GL patterns. To better illustrate the proposed method, Figure 10a shows the intensities of three phase-shift patterns at the reference plane, while Figure 10b shows the intensities of three nGC patterns. Figure 10c shows the codewords determined from Equations (17)–(19). Figure 10d shows the wrapped phase calculated from Equation (6). By looking up the position of the codeword in Table 1, the fringe order for each pixel can be determined as shown in Figure 10e. Then based on Equation (7), the absolute phase can be obtained as shown in Figure 10f. The 2π discontinuities are removed, and the absolute phase is continuous.

**Figure 11:**
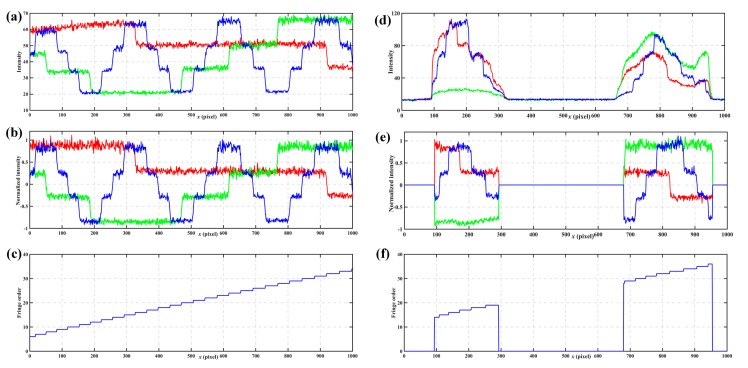
One cross-section of the *n*GC method. (**a**) *n*GC patterns without normalization of the reference plane, and (**b**) the same patterns after normalization, (**c**) fringe orders calculated from the normalization patterns for the reference plane, (**d**) *n*GC patterns without normalization of the measured objects, and (**e**) the same patterns after normalization, and (**f**) fringe orders calculated from the normalization patterns for the measured objects.

The first experiment shows the process of the *n*GC method. Figure 11a shows a cross-section of the reference plane without normalization. Figure 11b shows the same section of the reference plane after normalization. Because the surface contrast remains nearly the same in the reference plane, the intensity normalization shows little improvement. Figure 11c shows fringe orders calculated from the normalization patterns for the reference plane. Figure 11d shows a cross-section of the tested objects without normalization. Figure 11e shows the same cross-section of the tested objects after normalization, which illustrates significant differences compared to Figure 11d. It was hard for us to extract the codes from Figure 11d, but there was no difficulty in Figure 11e. Thus, intensity normalization can eliminate the influences of ambient light and surface contrast, which make the *n*GC more robust. Moreover, there is no fringe order error in Figure 11c,f, which also shows the robustness of the *n*GC method.

**Figure 12:**
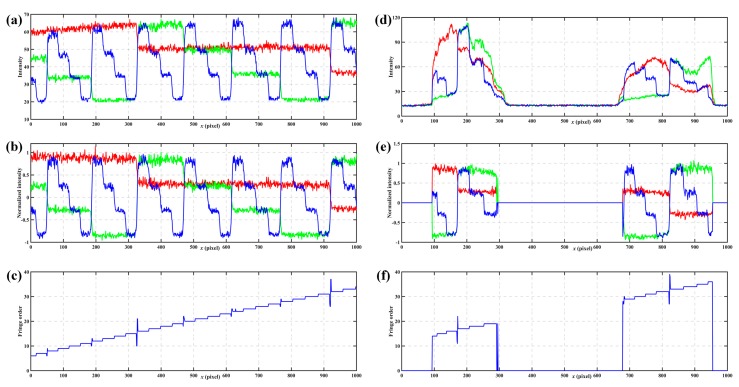
One cross-section of the *n*GL method. (**a**) *n*GL patterns without normalization of the reference plane, and (**b**) same patterns after normalization, (**c**) fringe orders calculated from the normalization patterns for the reference plane, (**d**) *n*GL patterns without normalization of the measured objects, and (**e**) same patterns after normalization, and (**f**) fringe orders calculated from the normalization patterns for the measured objects.

The next experiment shows the process of the *n*GL method. Figure 12a shows one cross-section of the reference plane without normalization. Figure 12b shows the same section of the reference plane after normalization. Figure 12c shows fringe orders calculated from the normalization patterns for the reference plane. Obviously, some sharp peaks occur in some of the code boundaries which will lead to the absolute phase errors. Figure 12d shows a cross-section of the tested objects without normalization. Figure 12e shows the same cross-section of the tested objects after normalization. Also, intensity normalization can eliminate the influences of ambient light and surface contrast. Figure 12f shows the fringe orders calculated from the normalization patterns for the measured objects. As with Figure 12c, some sharp peaks occur at the edge of some fringes in Figure 12f which leads to absolute phase errors. Comparing the results of the *n*GL method with the previous *n*GC method, the *n*GC method shows less errors happening, and therefore, be more robust in fringe order calculations.

**Figure 13:**
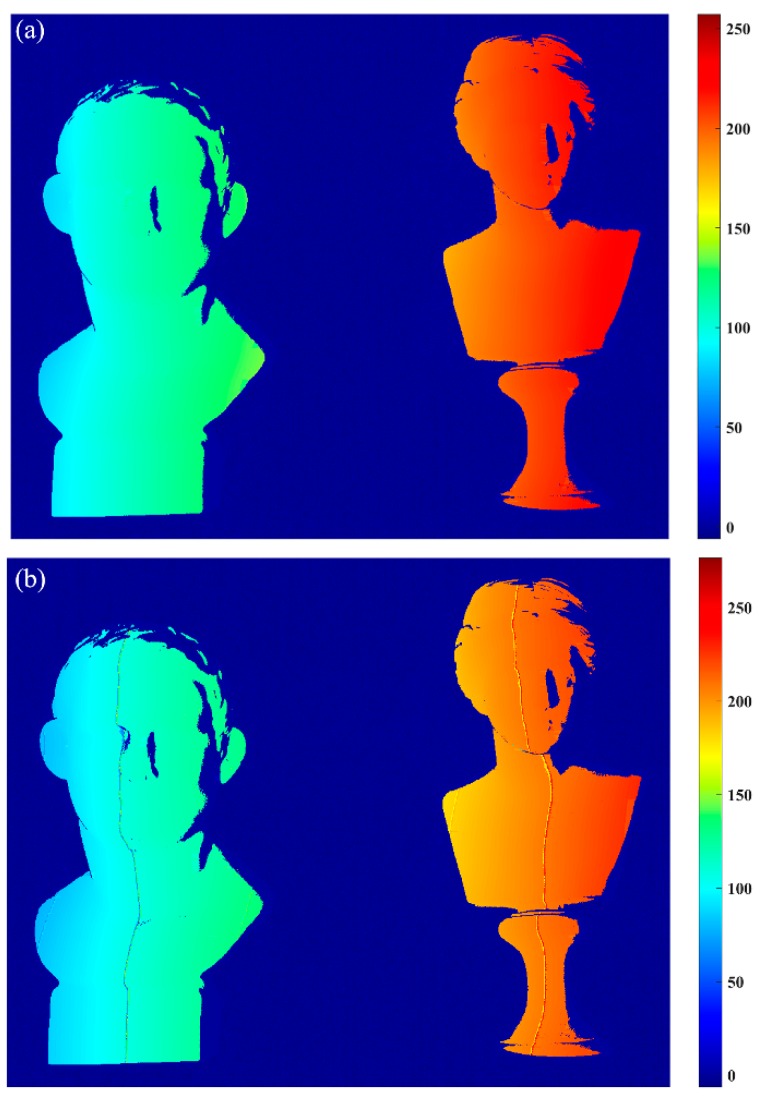
The absolute phase maps. (**a**) *n*GC method, (**b**) *n*GL method.

**Figure 14:**
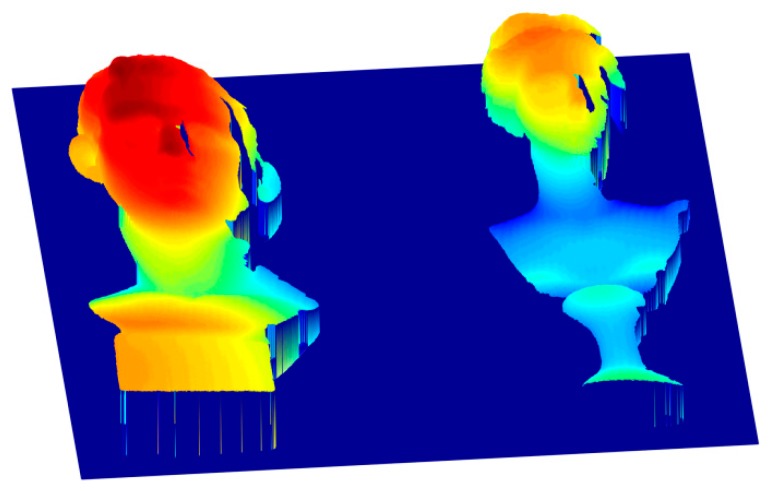
The 3D shape measurement result using the proposed method after correction.

Figure 13 shows the two different results according to the framework proposed in this paper, and the only difference is that the former one uses the *n*GC method, and the latter uses the *n*GL method. Figure 13a shows the absolute phase map using the *n*GC method, which has no obvious mistakes. However, the absolute phase map obtained from the *n*GL method, shown in Figure 13b, shows some obvious errors at the fringe boundaries because the blurred pattern images caused by the defocus effects of the projector or the camera may lead to incorrect codewords. The results confirm that the proposed method can be performed better than the *n*GL method. Finally, we present the 3D shape measurement resulting from the *n*GC method after correction in Figure 14.

## 5. Conclusions

This paper presents a modified gray-level method for absolute phase retrieval. The proposed method can generate many more codewords than the common *b*GL method, with *n* > 2 intensity values. An intensity normalization procedure, which takes the average intensity and modulation of phase-shift patterns into account, is developed to deal with the problems caused by ambient light and surface contrast. Compared with the *n*GL method, the *n*GC method can reduce the codeword detection errors and then improve the phase unwrapping. Both simulation and real experiments demonstrate that the proposed method is reliable and applicable.

## Figures and Tables

**Table 1:** The designed codewords.

***C_3_***	4				3				2				1			
***C_2_***	4	3	2	1	1	2	3	4	4	3	2	1	1	2	3	4
***C_1_***	4321	1234	4321	1234	4321	1234	4321	1234	4321	1234	4321	1234	4321	1234	4321	1234
